# Phenotypic Evidence of T Cell Exhaustion and Senescence During Symptomatic *Plasmodium falciparum* Malaria

**DOI:** 10.3389/fimmu.2019.01345

**Published:** 2019-06-18

**Authors:** Augustina Frimpong, Kwadwo Asamoah Kusi, Dennis Adu-Gyasi, Jones Amponsah, Michael Fokuo Ofori, Wilfred Ndifon

**Affiliations:** ^1^Department of Biochemistry, Cell, and Molecular Biology, West African Centre for Cell Biology of Infectious Pathogens (WACCBIP), University of Ghana, Accra, Ghana; ^2^Immunology Department, Noguchi Memorial Institute for Medical Research, College of Health Sciences, University of Ghana, Accra, Ghana; ^3^African Institute for Mathematical Sciences, Cape Coast, Ghana; ^4^Kintampo Health Research Centre, Kintampo, Ghana; ^5^African Institute for Mathematical Sciences, University of Stellenbosch, Cape Town, South Africa

**Keywords:** malaria, *Plasmodium falciparum*, T-cell, exhaustion, immune senescence, PD-1, CTLA-4, CD57

## Abstract

T cells play significant roles during *Plasmodium falciparum* infections. Their regulation of the immune response in symptomatic children with malaria has been deemed necessary to prevent immune associated pathology. In this study, we phenotypically characterized the expression of T cell inhibitory(PD-1, CTLA-4) and senescent markers (CD28(-), CD57) from children with symptomatic malaria, asymptomatic malaria and healthy controls using flow cytometry. We observed increased expression of T cell exhaustion and senescence markers in the symptomatic children compared to the asymptomatic and healthy controls. T cell senescence markers were more highly expressed on CD8 T cells than on CD4 T cells. Asymptomatically infected children had comparable levels of these markers with healthy controls except for CD8+ PD-1+ T cells which were significantly elevated in the asymptomatic children. Also, using multivariate regression analysis, CTLA-4 was the only marker that could predict parasitaemia level. The results suggest that the upregulation of immune exhaustion and senescence markers during symptomatic malaria may affect the effector function of T cells leading to inefficient clearance of parasites, hence the inability to develop sterile immunity to malaria.

## Background

Clinical malaria is a disease of public health importance due to its associated morbidity and mortality (1). With the emergence of drug-resistant parasites and insecticide resistant vectors, there is a need to develop effective interventions (2–4). Despite promising results of candidate vaccines in naïve individuals, comparatively poorer responses are observed in people in endemic areas (5, 6), indicating that much effort needs to be focused on understanding host factors associated with the development of immunity, especially in malaria-endemic areas. Blood stage infection with malaria parasites may either result in asymptomatic malaria, uncomplicated malaria, or proceed to complications such as severe malaria anemia or cerebral malaria. Repeated exposure to parasites usually results in the acquisition of anti-disease immunity which is characterized by the absence of clinical symptoms, yet with susceptibility to the infection. This suggests that the naturally induced immune response generated against *P. falciparum* may not always be potent enough to eradicate the infection. Therefore, malaria vaccines that can protect against symptomatic disease and possibly also eliminate infections remain a global health priority.

Lymphocytes, including T cells, play a significant role in the generation of protective malaria-specific responses (7), and their mechanism of action may either be by controlling or decreasing parasitemia (8) or by exacerbating the infection promoting parasitemia (9). However, looking at natural infections it can be presumed that the inability to eliminate *P. falciparum* malaria may be associated with immune dysfunction resulting from the expression of markers that negatively regulate T cell activity or result in their ineffective response. These may lead to the exhaustion of T cells, which has been well-described in viral infections including HIV and hepatitis B (HBV) (10, 11) as well as in protozoan infections like Toxoplasmosis and Leishmania (12, 13).

In malaria, work in both human and murine models has reported the upregulation of immune inhibitory markers such as T-cell immunoglobin and mucin domain-3 (TIM-3), lymphocyte-activation gene-3 (LAG-3) and programmed cell death-1 (PD-1) during acute infections (14, 15). These have been shown to affect not only the effector functions of T cells including cell proliferation and cytokine production but also antibody generation by B cells (16). Specifically, PD-1 has been associated with decreased cytokine production and proliferation in T cells as well as enhancing disease progression, whereas CTLA-4 has been associated with T cell anergy and establishment of immunological tolerance (17, 18). Furthermore, it has been shown that the dysfunctional nature of exhausted T cells in murine models of malaria can be reversed by blockage of these receptors as this enhances effective parasite clearance and acquisition of immunity (16, 19).

In addition to immune exhaustion, infectious pathogens such as Cytomegalovirus (CMV) and Human Immune deficiency virus (HIV) have been associated with accelerated aging of the body's immune defense system through the upregulation of CD57, a classical marker for immune senescence (20, 21). CD57 is a terminally differentiated marker found on some cell subsets including T cells (22–24). Naïve T cells express CD28, a co-stimulatory molecule that provides signaling for T cell activation) after antigen recognition and this may bind to B7 proteins to provide co-stimulatory signals (25, 26). However, repeated T cell activation is associated with the progressive loss of CD28, a characteristic of memory or terminally differentiated cells, and the corresponding upregulation of CD57 (27–29). These senescent cells are characterized by shortened telomeres, replicative senescence, loss of CD27 resulting in a low proliferative capacity of the cells (30), eventually, leading to an inability to eradicate an infection. Importantly, the expression of CD57 is associated with repeated antigen stimulation (31) which was identified to accurately predict replicative senescence (22). In addition, CD57 expression on CD28- T cells has been shown to differ from the normal aging T cell phenotypes (CD28-CD57+, similarly observed in CMV) (31, 32) found in HIV infections (33).

Cellular aging has been described in wild birds chronically infected with malaria (34). Interestingly, a recent study reported evidence of cellular aging in travelers with single acute *P. falciparum* infections, characterized by decreased telomerase activity and increased levels of CDKN2A, a molecular marker associated with cellular aging (35). Nevertheless, it remains to be elucidated if frequent exposure to malaria is associated with increased expression of markers of T cell senescence in endemic areas. Here, we determined the expression profile of inhibitory or exhaustive, and immune-senescence markers on both CD4+ and CD8+ T cells. We characterized the expression of PD-1, CTLA-4, CD28 and CD57 markers in children with symptomatic malaria, asymptomatic malaria and healthy controls. In addition, we also determined the impact of these T cell phenotypes on parasitaemia and inflammation (using the platelet-to-lymphocyte ratio).

## Materials and Methods

### Ethics Statement

The study protocols were approved by the Institutional Review Board of the Noguchi Memorial Institute for Medical Research at the University of Ghana. All participants were children and informed consent was obtained from parents or guardians and assent properly received from the children before they were enrolled in the study. All methods were performed in accordance with the relevant guidelines and regulations.

### Study Subjects

A total of 57 children within the age ranges of 1–12 years were recruited for the study, consisting of healthy children with no *P. falciparum* infections (*n* = 17), children with asymptomatic *P. falciparum* malaria (*n* = 18) and children with clinical malaria (*n* = 22) who were recruited from the Asutsuare and the Paakro sub-districts which are hyper-endemic areas for malaria transmission in Ghana. A volume of 5 ml of venous blood was collected from all study participants after recruitment. Parasites were identified using Giemsa stained thick and thin blood films. Clinical cases of malaria recruited from the health centers were defined by a history of fever within 24 h of health center attendance and presence of parasitaemia. For clinical cases, we collected venous blood samples from the children before anti-malarial treatment, based on the nationally recommended guidelines. Asymptomatic cases were recruited from the community and were defined by the presence of parasitaemia, absence of fever and no signs or symptoms of the disease. Healthy children, also recruited from the community were selected based on the absence of parasitaemia, fever and no signs or symptoms of the disease.

### Peripheral Blood Mononuclear Cells (PBMC) and Plasma Isolation

Isolation of PBMCs was performed by density gradient centrifugation using ficoll paque. After isolation, PBMCs were enumerated and cryopreserved in fetal bovine serum with 10% dimethyl sulfoxide. PBMCs were kept at −80°C overnight and subsequently transferred to liquid nitrogen until required for the experiment.

### Flow Cytometry Analysis

PBMCs were retrieved, thawed and washed. The viability was measured by the trypan blue dye exclusion method and cells with viability > 95% were used in the assay. All the antibodies were purchased from BD except anti-human FOXP3 fluorochrome-conjugated antibody (Biolegend). After washing, the cells were extracellularly stained with the following antibodies: anti-CD3-(APC H7), anti-CD4 (BUV 395), anti-CD8 (PerCP Cy5.5), anti-CD28 (APCR700), anti-CD57 (FITC) and anti-PD-1 (BUV737) on ice for 30 min. The cells were washed, fixed and permeabilized using FOXP3 buffer set (BD) according to manufacturer's instructions and intracellularly stained for FOXP3 (PE) and CTLA-4 (APC) on ice for 40 min. We gated for T cells by CD3, CD4, and CD8 lineage markers. Gates for inhibitory and senescence markers were defined using fluorescence minus one controls (Figure S1). Cells were acquired on a BD LSR Fortessa II-X20 cytometer. Data were compensated and analyzed using Flowjo V10 software (Tree Star, San Carlos, CA).

### Statistical Analysis

Data analyses were performed with R-studio for statistical analysis (version 2) and the GraphPad Prism version 6.01 (GraphPad Software, Inc.). For comparing the markers of T cells among the three study populations, the Kruskal-Wallis test with a Dunn's *post hoc* test for multiple comparisons was used. Spearman's rank correlation was used to determine associations between markers. Principal component analysis (PCA) was conducted to identify and visualize significant features of T cell phenotypes (degree of variation) that can cluster our study populations by considering all phenotypes measured. PCA which is an unsupervised learning algorithm provides dimensions (linear combinations) along which the data are separable and reduces the noise associated with data whilst increasing its robustness. PCA was used since it reduces the data set to a small set of patterns and retain the significant features that are responsible for variation (separating the data into clusters). Multiple linear regression models with likelihood ratio test were also used to investigate the association between parasitaemia or inflammation and the measured cellular markers. Statistical significance was set at *P* < 0.05.

## Results

### Clinical Characteristics of the Study Participants

The study was approved by the Noguchi Memorial Institute for Medical Research Institutional Review Board. This was a cross-sectional study in which we recruited 57 children in the age range of 1–12 years. The participants included 22 symptomatic children, 18 asymptomatic children and 17 healthy controls (Table 1). The sexes of the children were comparable amongst the study groups (*p* < 0.05). Healthy children were older than the asymptomatic(*p* < 0.05) and symptomatic children (*p* < 0.05). Levels of parasitemia mirrored the intensity of infection, with symptomatic children having a higher parasite load compared to the asymptomatic children (*p* < 0.001). Hemoglobin levels were significantly decreased in the symptomatic children in comparison to the asymptomatic children (*p* < 0.05). Even though, the lymphocyte count was not significantly different amongst the study groups, they also mirrored the intensity of infection (p>0.05). We found the granulocyte count to be comparable amongst the study groups (*p* > 0.05). Also, the platelet-to-lymphocyte ratio (PLR) was found to be comparable between the healthy controls and asymptomatic groups (*p* < 0.05) but higher than the symptomatic group (*p* < 0.05).

**Table 1:** Clinical characteristics of the study participants.

**Characteristics**	**Control**	**Asymptomatic**	**Symptomatic**	***P*-values**
	**(C)**	**(A)**	**(S)**	
Participants	*n* = 17	*n* = 18	*n* = 22	
Age (IQR), years	9(8–11)	7(4.5–9)	6(4.8–7)	0.0087^a^
Female (%)	52.94	44.44	50	0.8765^b^
Hemoglobin, g/dl^#^	11.5(0.994)	12.7(1.234)	10.7(3.025)	0.0402^c^
Parasitemia (IQR), μl	NA	845(260.7–3812)	13973(7238–58764)	0.0009^d^
Granulocytes (10^9^/L)^#^	3.353(1.335)	3.069(2.115)	5.041(3.310)	0.0518^c^
Lymphocytes (10^6^/L)	2.9(2.5–3.6)	2.1(1.2–3.45)	1.9(1.3–3.9)	0.0889^a^
Platelets (10^9^/L)	305(237–356)	223(193–280)	101(61–198)	>0.0001^a^
PLR(IQR)^&^	95.31(82.79–133.9)	70.97(27.52–205.3)	53.54(31.19–95.31)	0.0345^a^

*IQR, interquartile range*.

a *Kruskal Wallis test*.

b *Chi-square test*.

c *One-way ANOVA*.

d *Mann-Whitney U-test*.

# *Mean(Standard deviation)*.

& *(C = 17, A = 16, S = 19)*.

### Increased Expression of PD-1 and CTLA-4 Markers on T Cells in Children With Symptomatic *P. falciparum* Malaria

**Figure 1:**
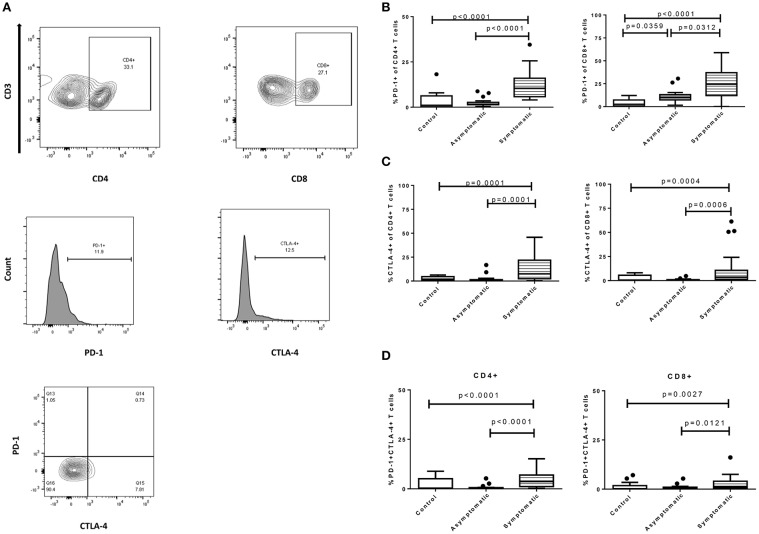
Expression profile of exhaustion and regulatory markers on CD4+ and CD8+ T cells among symptomatic malaria patients (*n* = 22), asymptomatic malaria(*n* = 18) and healthy children (*n* = 16). **(A)** The gating strategy to identify expression levels of PD-1 and CTLA-4 markers. The expression profile comparing levels of **(B)**. PD-1, **(C)**. CTLA-4, **(D)**. PD-1 and CTLA-4 double-positive markers, on both CD4+ and CD8+ T cells across the study subjects. Levels of expression were compared using the Kruskal-Wallis with Dunn's test to correct for multiple comparisons. The data are presented as box plots with inter-quartile ranges. The 10th and 90th percentiles are presented as whiskers with medians indicated by the bold horizontal lines across the boxes. *P* < 0.05 were considered statistically significant.

We first investigated the expression of the inhibitory markers PD-1 and CTLA-4 on T cells (Figure 1A). The expression levels of PD-1 were significantly upregulated in the symptomatic children compared to the asymptomatic (*p* < 0.0001) and healthy groups (*p* < 0.0001) for the CD4+ T cells (Figure 1B). Levels of PD-1 in the asymptomatic children and healthy children were comparable. Similarly, CD8+PD-1+ T cells were upregulated in children with symptomatic malaria compared to asymptomatic (*p* = 0.0312) and uninfected controls (*p* < 0.0001). Nevertheless, the expression of PD-1 on CD8+ T cells was increased significantly in the asymptomatic children compared to the healthy controls (*p* = 0.0359). Of note, the levels of PD-1 were higher in CD8+ T cells compared to the CD4+ T cells in all study groups. Also, the expression levels of CTLA-4 on CD4+ T cells were increased significantly in the symptomatic children compared to the asymptomatic (*p* < 0.001) and healthy controls (*p* < 0.05) whereas comparable levels of expression were found between asymptomatic children and healthy controls (Figure 1C). This trend was the same for the levels of CTLA-4 expression on CD8+ T cells among the study groups: symptomatic children had increased levels compared to asymptomatic (*p* < 0.001) and healthy children (*p* < 0.05).

Next, we assessed the expression of PD-1 and CTLA-4 double-positive markers on both T cell subsets. The symptomatic children had significantly higher levels of PD-1 and CTLA-4 double positive markers on CD4+ T cells in comparison to the asymptomatic children (*p* < 0.0001) and healthy controls (*p* = 0.0091). Similarly, levels on CD8+ T cells were higher in symptomatic children compared to the asymptomatic children (*p* = 0.0121) and healthy controls (*p* = 0.0098) (Figure 1D). In all, levels of PD-1 and CTLA-4 double positive markers between the asymptomatic children and healthy controls were comparable and not significantly different.

The significant levels of inhibitory markers observed in children with symptomatic malaria may be related to the inadequacy of effector functions in clearing parasitemia.

### Symptomatic *P. falciparum* Infection Is Associated With the Upregulation of T-Cell Senescence Markers

**Figure 2:**
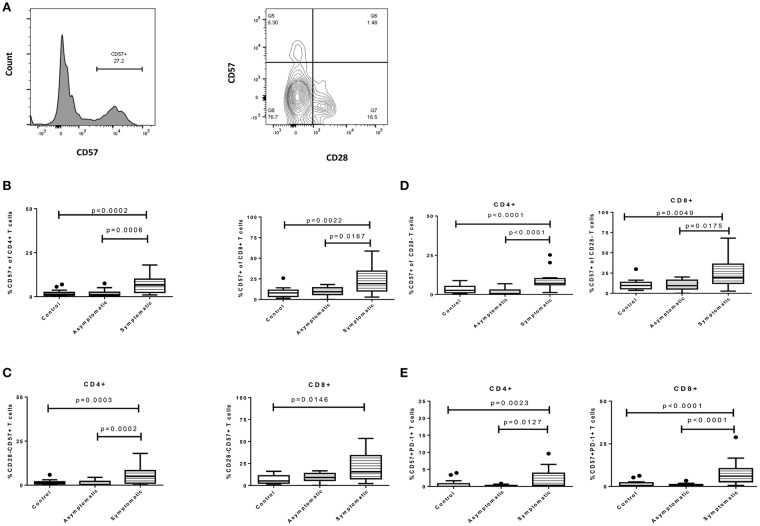
Symptomatic *P. falciparum* infection is associated with the upregulation of T-cell senescence markers. The expression profile of immune senescence markers on CD4+ and CD8+ T cells among symptomatic malaria patients, asymptomatic malaria and healthy children. **(A)** The gating strategy for identifying CD57 and CD28 surface markers. The expression profile comparing levels of **(B)**. CD57, **(C)**. CD28-CD57+, **(D)**. CD57+ of CD28- and **(E)**. PD-1+CD57+ markers on both CD4+ and CD8+ T cells across the study subjects. The data are presented as box plots with inter-quartile ranges. The 10th and 90th percentiles are presented as whiskers with medians indicated by the bold horizontal lines across the boxes. Levels of expression were compared among the study population using the Kruskal-Wallis with Dunn's test to correct for multiple comparisons. *P* < 0.05 were considered statistically significant.

We also determined if symptomatic malaria may be associated with the biological aging of T cells, by measuring senescent markers using CD28 and CD57 and comparing the proportions with the asymptomatic and healthy groups. We first determined the proportions of T cells expressing CD57 which were found to higher on CD8+ T cells compared to the CD4+ T cells. This trend was similar in all 3 study groups (Figure 2). Levels of CD4+CD57+ T cells were significantly higher in children with symptomatic malaria compared to asymptomatic (*p* = 0.0006) and healthy controls (*p* = 0.0041). A similar trend was observed for the CD8+ T cell subsets where a significant difference was observed between symptomatic and asymptomatic children (*p* = 0.0167) and healthy controls (*p* = 0.0050) (Figure 2A). Secondly, we checked for the percentage expression of CD28-CD57+ T cells, a marker frequently associated with T cell aging in the elderly. Levels of CD28-CD57+CD4+ T cells were also increased in children with symptomatic malaria compared to children with asymptomatic infections (*p* = 0.0002) and healthy controls (*p* = 0.0064). In contrast, levels of the CD28-CD57+ marker on CD8+ T cells did not differ between the symptomatic children and asymptomatic children (*p* = 0.1115), but was increased in the symptomatic group compared to healthy controls(*p* = 0.0178) (Figure 2B).

Later, we gated on CD28- T cells and measured the expression of CD57 on CD28- T cells (CD57 of CD28- T cells) to determine if the expression levels may be similar or differ from what is observed in normal aging or HIV infections. We found that the percentage expression of CD57+ on CD28-CD4+ T cells remained increased in children with symptomatic malaria compared to children with asymptomatic infections (*p* < 0.0001) and healthy controls (*p* = 0.0261). Also, the percentage expression of CD57 on CD28- CD8+ T cells was significantly increased in children with symptomatic malaria compared to those with asymptomatic malaria (*p* = 0.0175) or healthy controls (*p* = 0.0147) (Figures 2B,C).

We further compared the expression of CD57 and PD-1 double-positive markers (commonly associated with increased apoptosis) on T cells in the study participants. We observed that CD4+ T cells expressing both CD57 and PD-1 were increased in children with symptomatic malaria compared to asymptomatic (*p* = 0.0127) and healthy controls (*p* = 0.0071; Figure 2D). This trend was similarly observed in the CD8+ T cells: levels of PD-1+CD57+CD8+ T cells were increased in children with symptomatic malaria in comparison to asymptomatic children (*p* < 0.0001) and healthy controls (*p* = 0.0001). Overall, T cells from symptomatic *P. falciparum* infected children showed phenotypic evidence of T cell senescence.

### CTLA-4 Is a Major Predictor of Parasite Load During *Plasmodium falciparum* Infection4

**Figure 3:**
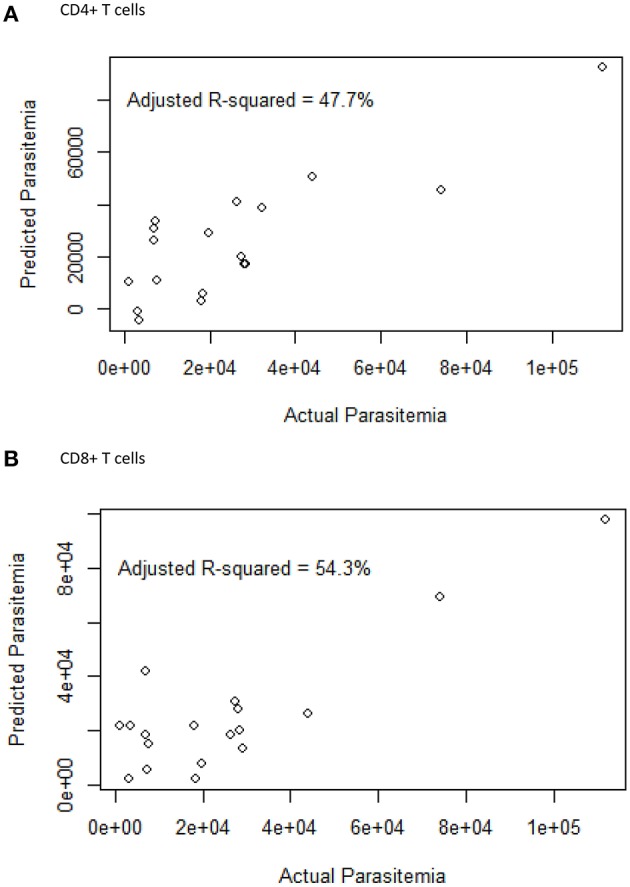
The relationship between the surface markers and parasitemia. A multiple linear regression plot. Actual parasitaemia levels were indicated on the x-axis with the predicted values of parasitaemia on the y-axis. The model was designed using 7 T cell phenotypes measured in **(A)** CD4 (PD-1, CTLA-4, CD57, PD-1CTLA-4, PD-1CD57, CD28-CD57+, CD57+ of CD28-) and **(B)** CD8 (PD-1, CTLA-4, CD57, PD-1CTLA-4, PD-1CD57, CD28-CD57+, CD57+ of CD28-) T cells from PBMCs obtained from the symptomatic children.

The effect of cellular markers on parasitemia and inflammation was investigated using multivariate regression analysis. We analyzed 7 T cell phenotypic markers; inhibitory (PD-1+, CTLA-4+, PD-1CTLA-4+) and senescence (CD57+, CD28-CD57+, CD57+ of CD28-, PD-1+CD57+)each on both CD4+ and CD8+ T cells to determine if any of these markers could predict parasitaemia or inflammation (PLR). We defined inflammation by the ratio of platelet-to-lymphocyte count (PLR) (36–38). Before the multivariate analysis, we initially performed a correlation analysis to determine if any of the phenotypes may be significantly associated with PLR or parasitaemia. The proportions of PD-1 (*r* = −0.65, *p* < 0.01) and CTLA-4 (*r* = −0.506, p < 0.05) were inversely correlated with PLR (Figure S4) but positively correlated with parasitaemia (for PD-1, *r* = 0.4631, *p* < 0.05; CTLA-4, *r* = 0.4831, *p* < 0.05). However, using the multiple linear regression model and performing a likelihood ratio test, expression levels of CTLA-4 on both CD4+ and CD8+ T cells were found to significantly predict the level of parasitemia in the symptomatic children (Figure 3; Tables 2, 3). Likewise, the levels of CD8+CD28-CD57+ and CD57 on CD8+CD28- T cells could significantly explain some of the variation observed in parasitemia (Tables 2, 3). Even though all the coefficients from the regression analysis for the T cell phenotypes were inversely associated with inflammation, none could be a predictor of inflammation (Table S1).

**Table 2:** The association of inhibitory markers on CD4+ T cells and parasitaemia for the symptomatic malaria population.

**Covariate**	**Coefficient**	***p-*value**	**LR test (*p-*value)**
CTLA4	0.386	0.12	0.032^*^
PD1	0.453	0.25	0.11
PD1CTLA4	0.509	0.57	0.44
CD57	0.499	0.48	0.33
CD57–CD28+	0.434	0.2	0.075
PD1CD57	0.52	0.75	0.66
CD28–CD57+	0.503	0.51	0.37

* *p < 0.05*.

*Statistically significant values are highlighted in bold*.

**Table 3:** The association of inhibitory markers on CD8+ T cells and parasitaemia for the symptomatic malaria population.

**Covariate**	**Coefficient**	***p*–value**	**LR test (*p*-value)**
CTLA4	0.253	0.018^*^	0.0012^**^
PD1	0.525	0.26	0.12
PD1CTLA4	0.545	0.351	0.2
CD57	0.563	0.491	0.34
CD57+CD28-	0.447	0.098	0.023^*^
PD1CD57	0.582	0.792	0.72
CD28-CD57+	0.438	0.09	0.02^*^

*Regression coefficients and p-values were determined using a multiple linear regression model. The effect of each phenotype in predicting the degree of inflammation was adjusted using the other considered phenotypes. The likelihood ratio (LR) test was used to assess the significance of each variable's effect by comparing the adjusted model with another model in which the covariate is absent. P < 0.05 was considered statistically significant. Statistically significant values are highlighted in bold*.

* *p < 0.05*,

** *p < 0.01*.

On the other hand, among the asymptomatic malaria group, levels of CD4+PD-1+ and CD4+PD-1+CD57+ could predict and explain some of the variation observed in parasitemia (*p* < 0.05; *p* < 0.0001) whereas for CD8+ T cells, the expression of CTLA-4 (*p* < 0.001) and PD-1+CTLA-4+ (*p* < 0.0001) were good predictors of parasitemia (Figure S3; Table S2).

### Multivariate Analysis of T Cell Inhibitory and Senescent Markers

**Figure 4:**
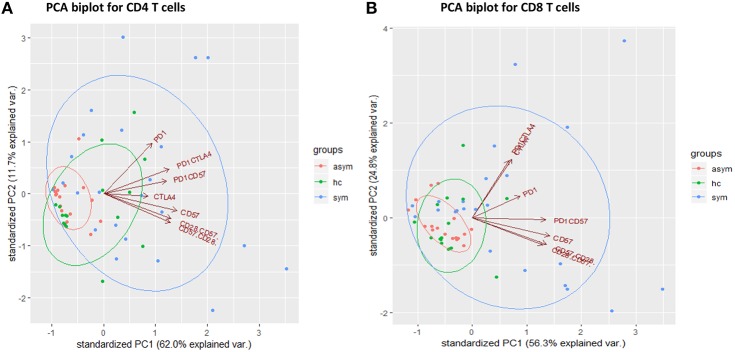
Principal component analysis of cellular markers determined in the healthy groups (*n* = 17), asymptomatic malaria (*n* = 18) and symptomatic malaria (*n* = 22), for **(A)** CD4 and **(B)** CD8 T cells. The green, red and blue symbols as well as ellipses denote the healthy, asymptomatic and symptomatic groups respectively. The red arrows indicate the cellular markers.

In order to identify significant immunological signatures (T cell phenotypes) that can explain the variation in our study population and separate our study population into clusters, we performed a principal component analysis (PCA). From the eigen values we obtained, we selected principal components that best explained the variations in the datasets. Components 1 and 2 for the CD4+ T cells accounted for 73.1% (62 and 11.7%, respectively) of the variation in data whereas, for CD8+ T cells, PC1 and PC2 accounted for 81.1% (56.3 and 24.8, respectively). From the plots, it can be observed that mostly the symptomatic group had higher PC values compared to the asymptomatic group. Using the entire datasets, the principal components clustered our population into three groups based on the frequencies of the phenotypes (Figures 4A,B). Also, from the CD8+ T cells, it can be observed that PC1 is associated with inhibitory markers located in the upper right quadrant whereas PC2 is associated with senescent markers, located in the lower right quadrant. In addition, the loadings of PD-1 and CD57 were significant for PC1 and PC2, respectively. Further analysis indicated the T cell phenotypes contributing to most of the variation for CD4 T cells were CTLA-4 and PD-1 whereas, for CD8 T cells, the important markers were PD-1, CD57 and CTLA4 (Figure S2).

### The Interrelationship Between the Cellular Inhibitory Markers

Next, we determined the interrelationship between the surface markers (including FOXP3) using partial correlation, a multiparametric correlation analysis that controls for confounding factors (Tables 4, 5). For instance, significant positive correlations for CD4 T cells were observed between FOXP3 and PD1, FOXP3 and CD57, PD-1CTLA-4 and CD57 (*p* < 0.05). Generally, significant correlations were all positively related.

**Table 4:** A partial correlation matrix with covariates between the cellular markers measured on CD4+ T cells for the symptomatic malaria population.

**CD4**	**CTLA4**	**PD1**	**PD1CTLA4**	**CD57**	**CD57+CD28-**	**PD1CD57**	**CD28-CD57+**	**FOXP3**
CTLA4	1							
PD1	−0.28392916	1						
PD1CTLA4	0.48244305	0.7425784^**^	1					
CD57	−0.09261014	−0.4476809	0.6287185^**^	1				
CD57+CD28-	0.28077369	0.1731718	−0.0862313	−0.06116931	1			
PD1CD57	0.36438345	0.1282666	−0.179054	−0.08438154	−0.2904794	1		
CD28-CD57+	0.03186114	0.3141221	−0.456166	0.91009944^****^	0.363051	0.1875853	1	
FOXP3	0.2481085	0.6309751^*^	−0.4845044	0.55752428^*^	−0.432212	−0.0890901	−0.3706132	1

*Significant correlations were determined using a permutation test. P < 0.05 were considered statistically significant*;

* *p < 0.05*,

** *p < 0.01*,

**** *p < 0.0001. Statistically significant values are highlighted in bold*.

**Table 5:** A partial correlation matrix with covariates between the cellular markers measured on CD8+ T cells for the symptomatic malaria population.

**CD8**	**CTLA4**	**PD1**	**PD1CTLA4**	**CD57**	**CD57+CD28-**	**PD1CD57**	**CD28-CD57+**	**FOXP3**
CTLA4	1							
PD1	0.4365899	1						
PD1CTLA4	0.8497852^****^	−0.267313	1					
CD57	−0.230747	0.0385105	0.136794	1				
CD57+CD28-	0.2025701	0.0277434	−0.1928562	0.6714082^**^	1			
PD1CD57	−0.2022586	0.720932^**^	0.1076203	0.0563088	0.14287562	1		
CD28-CD57+	0.1714043	−0.4517704	−0.0132945	0.2164709	0.46299502	0.2318686	1	
FOXP3	0.2319811	−0.3906262	0.1694245	0.0678562	−0.00587128	0.3006289	−0.29398143	1

*Significant correlations were determined using a permutation test. P < 0.05 were considered statistically significant*;

** *p < 0.01*,

**** *p < 0.0001. Statistically significant values are highlighted in bold*.

## Discussion

The upregulation of inhibitory and senescent markers on T cells has been associated with the impairment of effector T cell responses. In this study, we sought to identify T-cell immune signatures that may be associated with the development of symptomatic malaria. We analyzed the pattern of expression of co-inhibitory and senescent markers in children with symptomatic *P. falciparum* malaria, asymptomatic malaria and healthy controls. We found that the expression of these exhaustive and senescent markers was increased in children with symptomatic malaria compared to those with asymptomatic infections and healthy controls. Using multivariate regression analysis with likelihood ratio test, we found CTLA-4 to be a strong predictor of parasitemia levels. Also, none of the T cell phenotypes measured was a good predictor of inflammation, even though PD-1 and CTLA-4 were inversely correlated with inflammation. Using a principal component analysis, our study population was clustered into three groups based on the level of expression of the cellular markers. Further analysis revealed that, for CD4+ T cells, the levels of CD4+CTLA4+ and CD4+PD-1+ markers could explain the clustering pattern of the study groups, whereas for CD8+ T cells the important markers were CD8+PD-1+, CD8+CD57+ and CD8+CTLA-4+. In addition, we observed a lower platelet-to-lymphocyte ratio in the symptomatic malaria group probably resulting from the decreased platelets and lymphocytes counts that are associated with clinical malaria (39, 40).

The activation of T cells by pathogens leads to the induction of inhibitory receptors such as CTLA-4 and PD-1 (41). PD-1 and CTLA-4 are some of the well-characterized inhibitory receptors associated with the exhaustion of T cells (16, 42, 43). Levels of expression of the inhibitory markers PD-1 and CTLA-4 were upregulated in children with symptomatic malaria, confirming recent studies that have observed increased levels of PD-1 and CTLA-4 during acute infections (14, 44, 45), resulting in decreased production of cytokines (46). This, therefore, suggests that the increased frequency of inhibitory markers during clinical disease may alter the effector function of T cells.

It has been shown by Butler et al. (16) that levels of CD4+PD-1+ phenotypes correlate with parasitemia in clinical malaria. In this study, we found that CD4+PD-1+ and CD8+CTLA-4+ could also predict parasitemia levels in the asymptomatic malaria group. Importantly, in the symptomatic group, the expression of CTLA-4 was a major predictor in determining parasitemia load. This suggests that T cell exhaustion may induce tolerance which may promote parasitemia. Since both PD-1 and CTLA-4 are negative regulators of the immune response, their observed increase in symptomatic children may indicate that PD-1 and CTLA-4 contribute in regulating T cell activity or inflammation (14, 46). Interestingly, we found an inverse association between these inhibitory markers and inflammation, which is in line with previous observations in murine models that blockage of T cell inhibitory markers exacerbated the immune response, increased susceptibility to severe disease and decreased survival (47, 48). Furthermore, the strong association we observed between T cell exhaustion and clinical parameters such as parasitemia and inflammation suggests that T cell exhaustion plays a vital role in malaria pathogenesis.

We have previously shown that asymptomatic *P. falciparum* infections are characterized by a reduced frequency of regulatory T cells (49). Here, we hypothesized that asymptomatic infections may have reduced expression of inhibitory markers compared to symptomatic children. We found that the expression levels of inhibitory markers in asymptomatic and healthy controls were mostly comparable except for PD-1 which was increased in the *P. falciparum* infected groups compared to the healthy controls (43). It may indicate PD-1 expression may be driven by *P. falciparum* infections. This could also imply that continuous exposure to *P. falciparum* infections may render T cells to be defective in function. Unfortunately, we could not determine the functionality of these T cells to confirm this.

Immunosenescence is the aging of immune cells characterized by shortened telomeres and inability to replicate, (50) sensitivity to apoptosis and, phenotypically, the expression of CD57 (22). T cell senescent markers were more associated with CD8+ T cells compared to CD4+ T cells consistent with earlier reports that they accumulate at lower frequencies for CD4+ T cells in human periphery (51). Here, we found an increased expression of CD57+T cell subsets in children with symptomatic malaria (52). This may suggest that malaria accelerates the aging of the T cell pool. In addition, the increased expression of CD28-CD57+ marker observed in symptomatic children indicates a greater proportion of effector T cells in symptomatic malaria have a memory phenotype since these cell subsets have been described to be antigen experienced (22, 31, 51). It has previously been shown that PD-1+CD57+CD8+ T cells have increased sensitivity to apoptosis mediated by PD-1 (53). The observed increase in expression of CD57 and PD-1 double-positive markers on CD8+ T cells, therefore, indicates a greater risk of apoptosis of these cells in clinical malaria. Additionally, T cell aging has been well characterized in the elderly population, CMV and HIV infections (21, 30, 33). In contrast to CMV infections which leads to expansion of T cell senescent markers, HIV leads to a decrease in the expression of CD57 on CD28-CD8+ gated T cells, whereas levels of CD8+CD28-CD57+ remains unchanged. In our study, we observed that both the proportion of CD28- T cells that express CD57 were expanded in the symptomatic malaria group, suggesting that T cell aging in falciparum infections is more similar to that observed during CMV infections than in HIV infections. Together, these observed phenotypic changes might reduce the responsiveness of the T cell repertoire to *P. falciparum* antigens resulting in an impaired ability to eliminate parasitemia.

FOXP3 is an immune regulatory marker associated with preventing immunopathology during inflammation. Both FOXP3 and PD-1 have been shown to suppress host immune response. Importantly, *P. falciparum* infections has been reported to cause the induction of PD-1+CTLA4+ T cells that control T cell activity (14). In this study, the positive correlation observed between FOXP3 and PD-1 T cells as well as between PD-1 and PD-1CTLA4 T cells could indicate that these markers play complementary roles in mediating the increasing immune activation that is associated with symptomatic malaria. There are conflicting reports about the role of CD57+ T cells in clinical disease, with some reports describing them as immunosuppressive and others suggesting they exacerbate immune activity through IFNγproduction (31, 54). Here, we observed a significant positive correlation between CD4+CD57+ T cells and CD4+FOXP3+ T cells as well as between CD4+CD57+ and CD4+PD-1+CTLA-4+ T cell subsets. However, since we could not determine the functionality of the CD57 T cell subsets, we can only suggest that the positive relationship observed between CD4+CD57 and CD4+FOXP3+ as well as CD4+ PD-1+CTLA-4+ T cell subsets may indicate that CD4+CD57+ T cells play suppressive roles during clinical malaria. This further supports the view that *Plasmodium* infections induce immunosuppressive immune responses that enhance the development of tolerance to the parasite, a mechanism affecting the development of sterile immunity.

Also, the results from the principal component analysis may indicate that a selection panel of the considered markers may serve as a biomarker for identifying individuals with symptomatic disease. It may probably be used to predict the outcome or immune response to vaccination. These results provide a basis to perform functional assays to determine the impact of the considered markers on the acquisition of anti-disease immunity during *P. falciparum* infections, preferably in a longitudinal cohort.

Studies have reported a low ratio of platelet-to-lymphocyte count (PLR) as a marker for inflammation in various infectious diseases such as HBV (36) and HCV (37). In this study, even though none of our markers was a good predictor of inflammation as previously stated using the PLR, we show that symptomatic malaria is characterized by low ratio of platelet-to-lymphocyte counts, which is indicative of on-going inflammatory response. Nonetheless, additional studies are needed to determine the significance of other hematological markers of inflammation (such as the neutrophil to lymphocyte ratio) to ascertain their clinical relevance during symptomatic malaria.

This study had a number of limitations due to the cross-sectional nature. We could not determine the effect of anti-malarial treatment on the expression of these inhibitory and senescent markers since samples were taking before the initiation of treatment. Furthermore, we defined *P. falciparum* infections by microscopy which is not able to distinguish between microscopic and sub-microscopic infections. In addition, we could not determine the effect of these markers on T cell cytokine production since cytokine profile analysis was not performed.

Despite these shortcomings, this study shows evidence that the phenotypic defect of T cells during *P. falciparum* infections are more pronounced in clinical malaria and associated with higher expression of exhaustive and senescent markers compared to asymptomatic infections. CTLA-4 was a good predictor of parasitemia in both symptomatic and asymptomatic malaria groups. Also, using the platelet to lymphocyte ratio, none of the markers measured could predict inflammation. In addition, we observed that the aging phenotype of T cells in malaria infection is similar to that observed with normal aging and CMV infections. These may imply that the increased expression of these markers may be associated with the absence of sterile immunity to *P. falciparum* malaria.

## Ethics Statement

This study was carried out in accordance with the recommendations of the Noguchi Memorial Institute for Medical Research, ethical review committee with written informed consent from all subjects. All subjects gave written informed consent and assent in accordance with the Declaration of Helsinki. The protocol was approved by the Institutional Review Board of the Noguchi Memorial Institute for Medical Research at the University of Ghana (NMIMR-IRB CPN 096/15-16).

## Consent for Publication

All authors have read and agreed to the content of this manuscript and its publication upon acceptance.

## Author Contributions

AF and MO conceived the idea and designed the experiments. WN, MO, and KK supervised the work. AF performed the experiments in the study and was assisted by DA-G and JA. AF, KK, MO and, WN wrote the paper. All authors read and approved the final manuscript.

### Conflict of Interest Statement

The authors declare that the research was conducted in the absence of any commercial or financial relationships that could be construed as a potential conflict of interest.
