# Supplementary material for: Phenotypic Evidence of T Cell Exhaustion and Senescence During Symptomatic Plasmodium falciparum Malaria
Source: Front Immunol. 2019 Jun 18;10:1345. doi: 10.3389/fimmu.2019.01345 (PMC6611412; doi:10.3389/fimmu.2019.01345)
Supplement: Supplementary file 1 [file Data_Sheet_1.docx]

**Table S1**. The association between inflammation and inhibitory markers on CD4+ and CD8+ T cells for the symptomatic malaria population

| **CD4** |  |  |  | **CD8** |  |  |  |
| --- | --- | --- | --- | --- | --- | --- | --- |
| **Covariate** | **Coefficient** | **p value** | **LR test(p value)** | **Covariate** | **Coefficient** | **p value** | **LR test(p value)** |
| CTLA4 | -0.26 | 0.54 | 0.32 | CTLA4 | -0.572 | 0.97 | 0.95 |
| PD1 | -0.439 | 0.29 | 0.093 | PD1 | -0.572 | 0.96 | 0.95 |
| PD1CTLA4 | -0.224 | 0.69 | 0.45 | PD1CTLA4 | -0.598 | 0.74 | 0.62 |
| CD57 | -0.283 | 0.49 | 0.27 | CD57 | -0.58 | 0.85 | 0.77 |
| CD57CD28 | -0.239 | 0.59 | 0.39 | CD57CD28 | -0.578 | 0.87 | 0.8 |
| PD1CD57 | -0.313 | 0.43 | 0.21 | PD1CD57 | -0.572 | 0.96 | 0.94 |
| CD28CD57 | -0.317 | 0.43 | 0.21 | CD28CD57 | -0.571 | 1 | 1 |

We adjusted the effect of each phenotype in predicting the degree of inflammation (platelet-to-lymphocyte ratio) using the other markers measured. The likelihood ratio test was used to test for the significance of each variable on the model by comparing the adjusted model with another model for which the covariate is absent. P<0.05 was considered statistically significant. Both models were not significant.

**Table S2**. The association of between parasitemia and inhibitory markers on CD4+ and CD8+ T cells for the asymptomatic malaria population

| **CD4** |  |  |  | **CD8** |  |  |  |  |
| --- | --- | --- | --- | --- | --- | --- | --- | --- |
| **Covariate** | **Coefficient** | **p value** | **LR test(p value)** | **Covariate** | **Coefficient** | **p value** | **LR test(p value)** | |
| CTLA4 | 0.5443 | 0.306 | 0.1596 | CTLA4 | -0.08919 | **0.0002** | **<0.0001** |  |
| PD1 | 0.4268 | 0.0722 | **0.0135** | PD1 | 0.7092 | 0.313 | 0.1654 |  |
| PD1CTLA4 | 0.5626 | 0.418 | 0.2656 | PD1CTLA4 | 0.3793 | **0.0041** | **<0.0001** |  |
| CD57 | 0.5678 | 0.462 | 0.3116 | CD57 | 0.6717 | 0.14 | 0.0427 |  |
| CD57CD28 | 0.5702 | 0.484 | 0.3359 | CD57CD28 | 0.7229 | 0.455 | 0.3042 |  |
| PD1CD57 | 0.3701 | **0.0007** | **<0.0001** | PD1CD57 | 0.6939 | 0.22 | 0.0917 |  |
| CD28CD57 | 0.565 | 0.437 | 0.2849 | CD28CD57 | 0.6932 | 0.217 | 0.0894 |  |

We adjusted the effect of each phenotype in predicting the degree of inflammation (platelet-to-lymphocyte ratio) using the other markers measured. The likelihood ratio test was used to test for the significance of each variable on the model by comparing the adjusted model with another model for which the covariate is absent. P<0.05 was considered statistically significant. Statistically significant values are highlighted in bold.
