# Supplementary figures and images for: Phenotypic Evidence of T Cell Exhaustion and Senescence During Symptomatic Plasmodium falciparum Malaria
Source: Front Immunol. 2019 Jun 18;10:1345. doi: 10.3389/fimmu.2019.01345 (PMC6611412; doi:10.3389/fimmu.2019.01345)

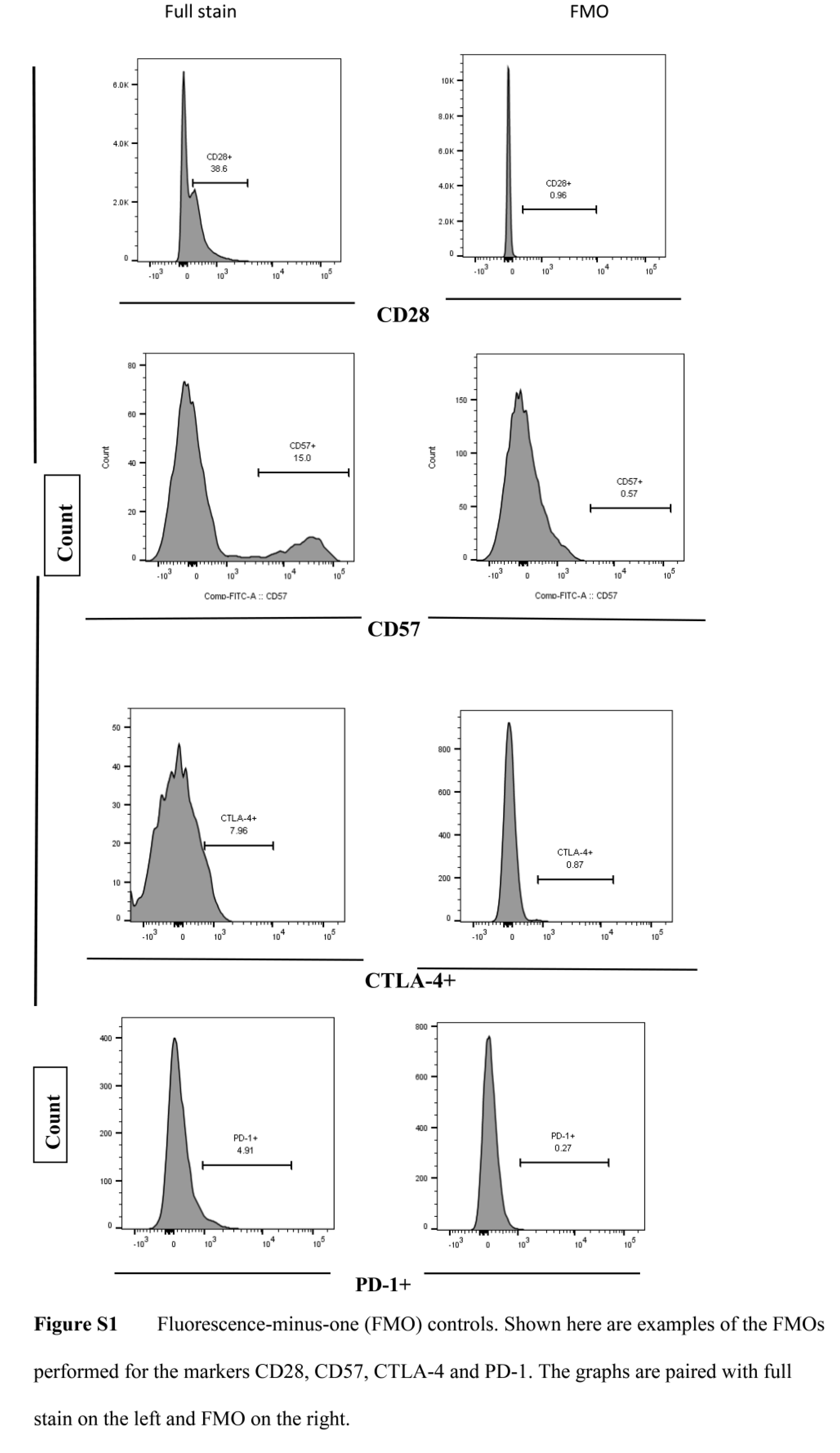

Supplement: Supplementary file 2 [file Image_1.JPEG]

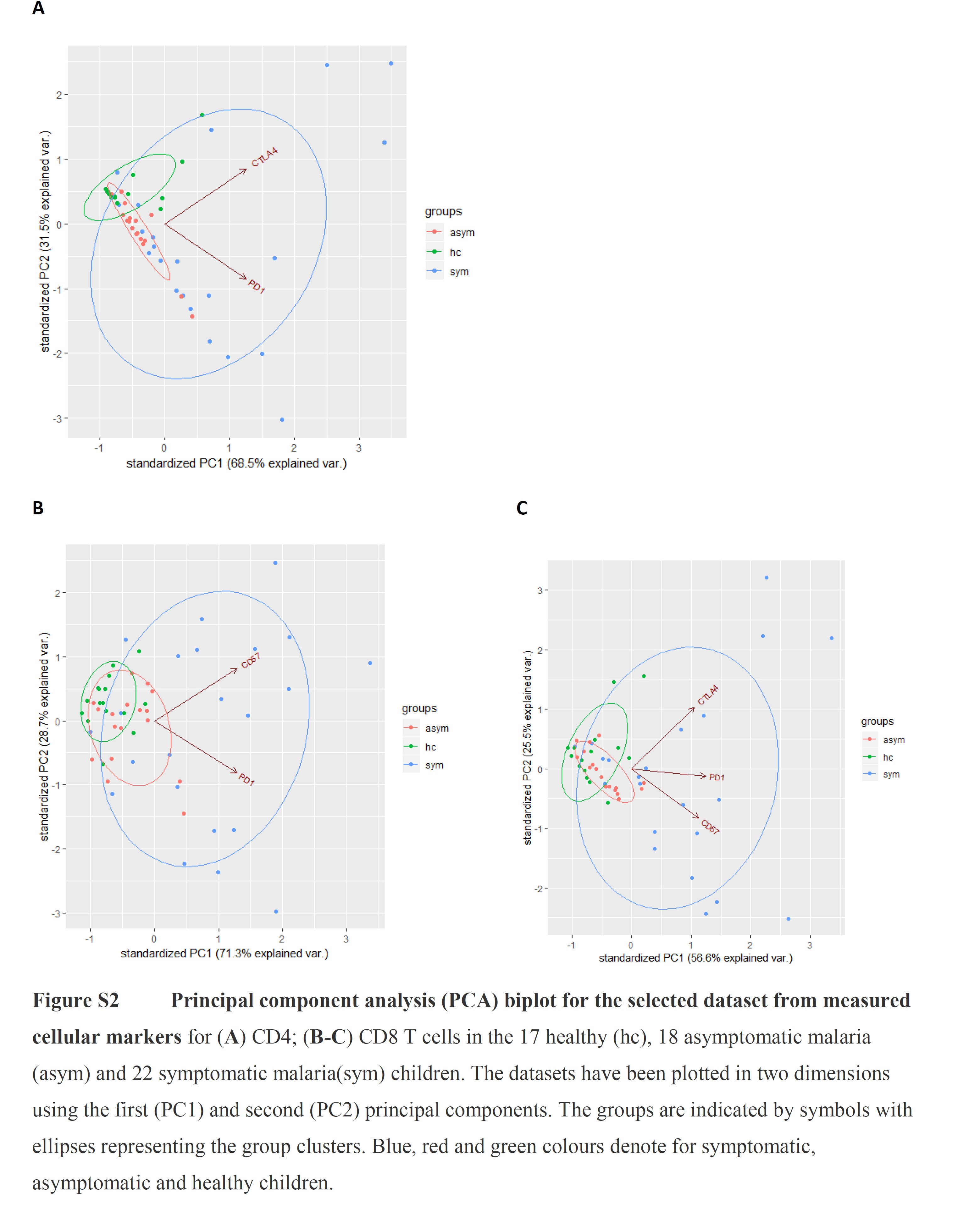

Supplement: Supplementary file 3 [file Image_2.JPEG]

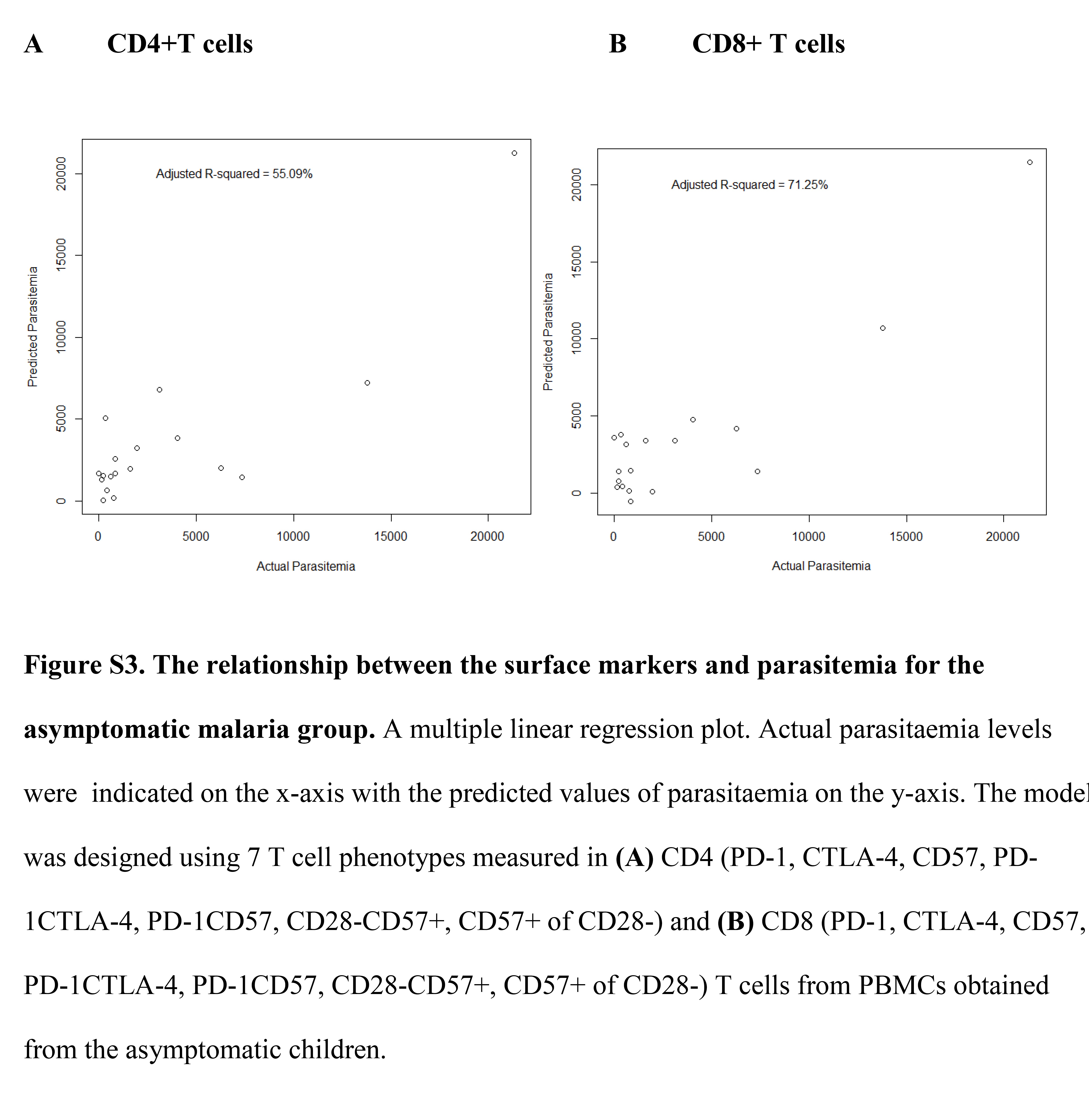

Supplement: Supplementary file 4 [file Image_3.JPEG]

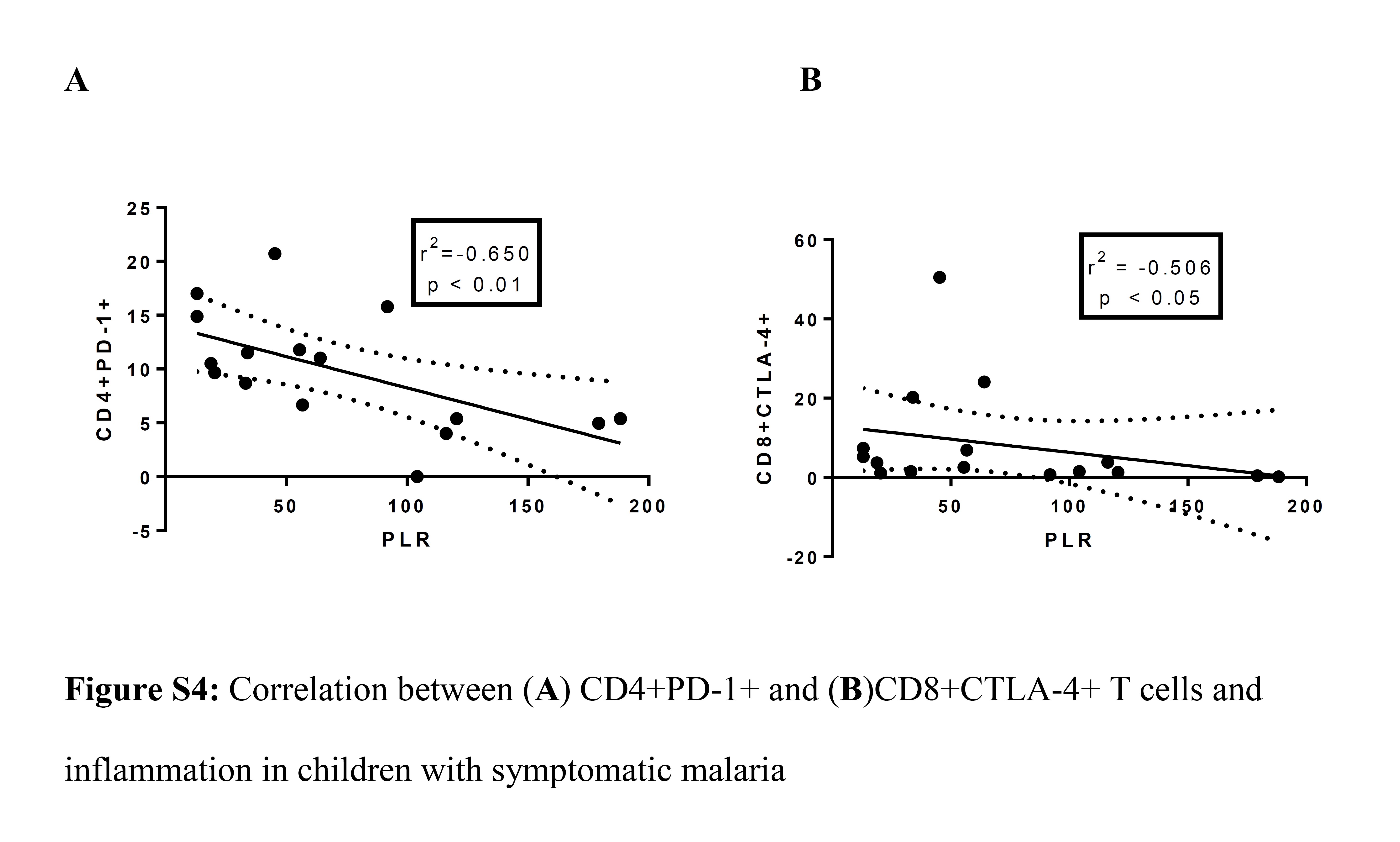

Supplement: Supplementary file 5 [file Image_4.JPEG]
